# Duplicate gene evolution and expression in the wake of vertebrate allopolyploidization

**DOI:** 10.1186/1471-2148-8-43

**Published:** 2008-02-08

**Authors:** Frédéric JJ Chain, Dora Ilieva, Ben J Evans

**Affiliations:** 1 Center for Environmental Genomics, Department of Biology, Life Sciences Building Room 328 McMaster University, 1280 Main Street West, Hamilton, ON, L8S 4K1, Canada; 2 Michael DeGroote School of Medicine – 5045, McMaster University, 1200 Main Street West, Hamilton ON L8N 3Z5, Canada

## Abstract

**Background:**

The mechanism by which duplicate genes originate – whether by duplication of a whole genome or of a genomic segment – influences their genetic fates. To study events that trigger duplicate gene persistence after whole genome duplication in vertebrates, we have analyzed molecular evolution and expression of hundreds of persistent duplicate gene pairs in allopolyploid clawed frogs (*Xenopus *and *Silurana*). We collected comparative data that allowed us to tease apart the molecular events that occurred soon after duplication from those that occurred later on. We also quantified expression profile divergence of hundreds of paralogs during development and in different tissues.

**Results:**

Our analyses indicate that persistent duplicates generated by allopolyploidization are subjected to strong purifying selection soon after duplication. The level of purifying selection is relaxed compared to a singleton ortholog, but not significantly variable over a period spanning about 40 million years. Despite persistent functional constraints, however, analysis of paralogous expression profiles indicates that quantitative aspects of their expression diverged substantially during this period.

**Conclusion:**

These results offer clues into how vertebrate transcriptomes are sculpted in the wake of whole genome duplication (WGD), such as those that occurred in our early ancestors. That functional constraints were relaxed relative to a singleton ortholog but not significantly different in the early compared to the later stage of duplicate gene evolution suggests that the timescale for a return to pre-duplication levels is drawn out over tens of millions of years – beyond the age of these tetraploid species. Quantitative expression divergence can occur soon after WGD and with a magnitude that is not correlated with the rate of protein sequence divergence. On a coarse scale, quantitative expression divergence appears to be more prevalent than spatial and temporal expression divergence, and also faster or more frequent than other processes that operate at the protein level, such as some types of neofunctionalization.

## Background

Gene duplication can catalyze the evolution of novel function by providing a respite from purifying selection [1]. The most common fate of a duplicated copy, however, is nonfunctionalization (pseudogenization), raising the question of how and why both copies of some duplicates manage to persist as functional entities. Interestingly, duplicate gene longevity is positively correlated with the scale of gene duplication – duplicate genes derived from whole genome duplication (WGD) typically persist for a longer period and evade pseudogenization at a higher frequency than those generated by segmental duplication [2-5]. Therefore it appears that mechanisms that promote duplicate gene persistence in polyploid genomes are either different from or more effective than those that operate on duplicated genes generated by segmental duplication. This is probably because mechanisms specific to polyploid genomes, such as stoichiometric requirements/genic balance, increase their longevity [6-9], whereas characteristics specific to segmental duplicates, such as incomplete coding regions and regulatory elements decrease theirs [10]. Furthermore, prezygotic isolating mechanisms could increase assortative mating within ploidy levels [11], facilitating speciation of polyploids and fixation of their duplicated genome in a new species. In clawed frogs, for example, second generation backcrossed hybrid females can produce a clutch comprised of fertile polyploid individuals of both sexes [12,13]. Sympatric speciation could be essentially instantaneous if these polyploid siblings interbreed and if reproductive incompatibilities exist between them and the lower ploidy parental species. In contrast, segmental duplicates begin as polymorphisms whose probability of fixation and time to fixation depend on genetic drift and natural selection [14].

If stoichiometry is important, then an incentive immediately exists to preserve unadulterated versions of both copies of duplicates generated by WGD. Duplicate genes could also persist without functional change after duplication if overexpression is advantageous [15,16], if there is selection against expression of a defective protein [17], or if neofunctionalized alleles were already segregating prior to duplication [18]. However, if neofunctionalizing mutations are rare or not very advantageous, or if population size is small, pre-duplication neofunctionalization is unlikely to be a common mechanism for duplicate gene persistence [18,19], although clearly it has occurred [20]. Duplication could also facilitate the resolution of conflicts that arise from gene sharing – when two distinct protein phenotypes arise from the same transcriptional unit – such as if an altered expression level is advantageous in one tissue but disadvantageous in another [21]. In duplicates generated via WGD by allopolyploidization, heterosis from interactions between diverged subgenomes could contribute to duplicate gene longevity without necessitating altered function after duplication [22].

An alternative explanation is that persistence of duplicates is triggered by genetic modification of one or both paralogs after duplication. For example, duplication could permit each copy of a multifunctional protein to specialize on a subset of the ancestral activities, thereby reducing pleiotropy [23,24]. Duplicates might also be preserved if each paralog degrades in a complementary fashion [25,26] or if one or both paralogs acquire novel function [1,27]. The post-duplication neofunctionalization model, for example, posits that one gene copy carries out the ancestral function(s), while the other one evolves neutrally and then acquires beneficial mutations by chance during the early stages of evolution [1]. Once new function is achieved, purifying selection is expected to dominate later stages of evolution. Neofunctionalization could occur with complete loss, partial degradation, or retention of ancestral function [28]. The duplication-degeneration-complementation model, also known as subfunctionalization, posits that after duplication each paralog degenerates in a complementary fashion such that the action of both is necessary to accomplish the full suite of ancestral activities [25,29]. Subfunctionalization could occur at the expression level through degeneration of paralogous expression profiles in a spatial, temporal, or quantitative dimension [25,29,30]. It could also occur at the protein level through complementary degeneration of different functional domains [25] or as a consequence of activity compromising substitutions [26]. The cellular location of expression also has an impact on protein function, and subcellular relocalization could facilitate or catalyze the evolution of unique functions in paralogs [31].

If genetic modification triggers the persistence of both paralogs, it must occur within a few million years after duplication or else one copy will likely become a pseudogene [6]. Moreover, the tempo of genetic modification after duplication may be dynamic, wherein changes that occur when the duplicate is young differ in frequency or nature from those that occur later on. After subfunctionalization or post-duplication neofunctionalization has occurred, for example, purifying selection is expected to increase. Additionally, some of these mechanisms for duplicate gene retention are not mutually exclusive and could operate concurrently or sequentially [28,32] and this could also be associated with temporal changes in functional constraints. To better understand the genetic basis of duplicate gene survival, it is therefore useful to consider their early stages of evolution separately from their later stages [5,6,33,34]. Comparison of young to old duplicates suggests that the rate of nonsynonymous substitutions is higher on average in younger duplicates [6,35,36]. This observation was interpreted as evidence of relaxed purifying selection immediately after duplication that was then followed by increased selective constraints as the duplicates aged. However, because pseudogenization rapidly transforms most young duplicates to singletons, it is not yet clear the degree to which evolution of young duplicates is indicative of the early stages of evolution of those exceptional duplicates that evade pseudogenization for dozens of millions of years.

**Figure 1:**
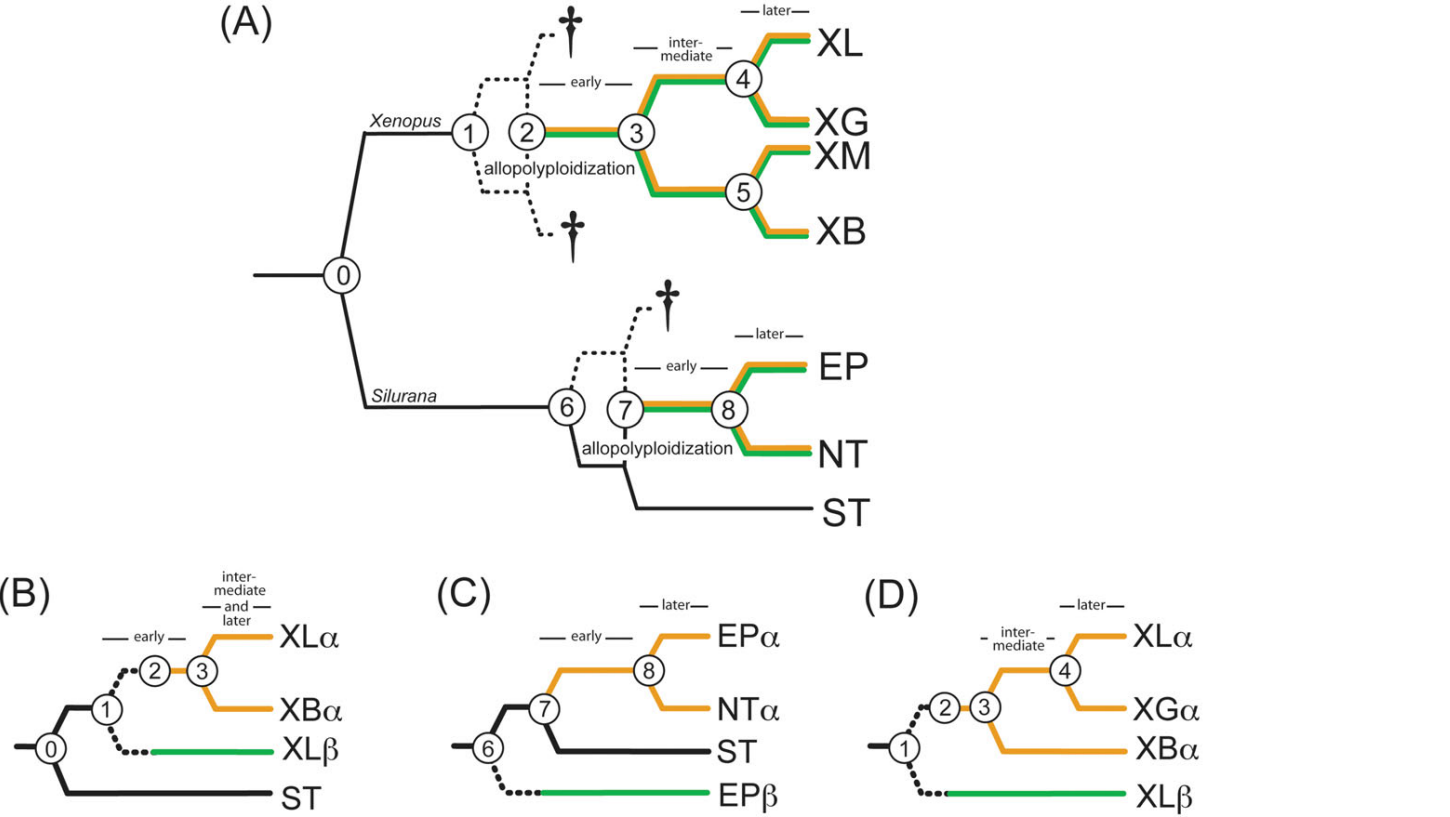
**Phylogenetic and genealogical relationships of species and paralogs in this study**. Phylogenetic relationships are depicted among species, orthologs, and paralogs of a diploid with 20 chromosomes, *S. tropicalis *(ST), two tetraploids with 40 chromosomes, *S. epitropicalis *(EP) and *S*. new tetraploid (NT), and four tetraploids with 36 chromosomes, *Xenopus laevis *(XL), *X. borealis *(XB), *X. gilli *(XG), and *X. muelleri *(XM). (A) Clawed frogs speciate by allopolyploidization and by regular speciation without a change in genome size. Allotetraploidization occurred independently in *Xenopus *and in *Silurana *and produced two paralogs in the resulting tetraploid ancestor – α and β – that are indicated as brown and green lineages respectively. After allopolyploidization, some of the diploid lineages probably went extinct, and this is indicated by a dagger. As a result of these extinctions, the portion of some paralogous lineages that evolved in a diploid, indicated as dashed lines, cannot be dissected apart from the portion that evolved in an allopolyploid. Numbered nodes indicate (0) divergence of the genera *Xenopus *and *Silurana*, (1) divergence of the diploid (2n = 18) ancestors of *Xenopus*, (2) allotetraploidization in *Xenopus*, (3) the first speciation event of the tetraploid ancestor of extant *Xenopus*, (4 and 5) more recent speciation events of *Xenopus *tetraploids, (6) divergence of the diploid (2n = 20) ancestors of *Silurana*, (7) allotetraploidization in *Silurana*, (8) speciation of a tetraploid *Silurana *without change in genome size. Sequences from individual paralogs were used to construct genealogies in order to compare (B) an early to a later stage of evolution after WGD in XLα, (C) an early to a later stage of evolution after WGD of EPα and (D) an intermediate to a later stage after WGD in XLα. Depending on the paralog for which data were obtained, sometimes NTα was considered in (C) or XBα was considered in (D).

To understand why so many duplicates persist after WGD, such as those that occurred in the ancestor of jawed vertebrates [37], teleost fish [2], and salmonid fish [38], additional information is needed about temporal dynamics in protein evolution and expression in the earliest stages of this type of genomic metamorphosis. In particular, we would like to dissect apart the molecular changes in the protein-coding region that occurred when persistent duplicates were young (an early stage of duplicate gene evolution) from those changes that occurred in the *same duplicates *after they became old (a later stage of duplicate gene evolution). Also of interest is the question of whether and how quickly paralogous expression profiles diverge after WGD. Polyploid clawed frogs (*Xenopus *and *Silurana*) are a useful model for studying early genetic events in vertebrate WGD because two independent instances of tetraploidization occurred fairly recently [32,39] and because subsequent speciation events occurred after both of these WGDs (Fig. 1A).

Previous studies have used this system to compare molecular evolution before and after WGD [32,40-42]. These studies indicate that purifying selection on *X. laevis *paralogs is relaxed compared to single-copy genes in the diploid species *S. tropicalis *[32,41,42], compared to single-copy orthologs in mammals [40,42], and compared to single-copy genes in *X. laevis *[42]. Using different statistical methods, independent tests on different genes recover evidence for asymmetric amino acid substitution in 4–6% of expressed paralogs in *X. laevis *[32,42]. We have used this system to explore duplicate gene evolution over different time intervals after WGD (tetraploidization), and to evaluate expression divergence of the resulting paralogs in *X. laevis*.

## Results

In *Xenopus *and in *Silurana*, because a tetraploid ancestor speciated, the timing of molecular changes that occurred after allopolyploidization can be dissected apart into two stages: an "early" stage of duplication – after allopolyploidization but before speciation of the tetraploid ancestor – and a "later" stage of duplicate gene evolution – after allopolyploidization and speciation of the tetraploid ancestor (Fig. 1). This permits the testing of alternative evolutionary scenarios of duplicate gene evolution. Moreover, the likelihood of sequence data can be quantified under a model with no change in the rate ratio of nonsynonymous to synonymous substitution (Ka/Ks ratio) before versus after tetraploid speciation, and it can be compared to the likelihood of an alternative model in which there is a different Ka/Ks ratio during these two stages of duplicate gene evolution. This analysis is not the same as a comparison of young to old duplicates, which involves comparing different genes that were duplicated at different times – instead it allows comparison of an early stage of evolution to a later stage of evolution of the *same *duplicates.

### Synonymous divergence

We collected and analyzed sequence data from fragments of hundreds of expressed paralogs from multiple species with an aim of teasing apart early from later mutations in the protein coding region of persistent paralogs generated by WGD (Fig. 1). In *Xenopus*, a concatenated analysis of 80,856 base pairs (bp) of expressed paralogs indicates that synonymous substitutions per synonymous site (Ks) between *X. laevis *paralogs (XLα and XLβ in Fig. 1B) is 0.2111, and Ks between the alpha paralogs of *X. laevis *and *X. borealis *(XLα and XBα in Fig. 1B) is 0.1393. This suggests that Ks between paralogs in the "early" stage of duplicate gene evolution is up to 0.0718, depending on the location of node 2 in Fig. 1B. Most synonymous divergence between paralogs therefore accumulated after tetraploidization in *Xenopus *(see Additional file 1), which occurred roughly 20 to 40 million years ago [32] or maybe more [39]. *Silurana *allotetraploids are about half as old [39].

### Rapid and persistent purifying selection after duplicate gene evolution

After allopolyploidization, these paralogs were rapidly (immediately or soon after WGD) subjected to strong purifying selection. The level of purifying selection, while relaxed relative to singletons [32,41], did not vary substantially between early and later stages of duplicate gene evolution.

More specifically, a combined analysis of thousands of codons from hundreds of expressed paralogs from *X. laevis*, a *X. borealis *ortholog, and a *S. tropicalis *ortholog, indicates that a more parameterized model of sequence evolution with a higher Ka/Ks ratio during the early stage of duplicate gene evolution than the later stage is not preferred (P = 1.00, Table 1, Fig. 1B). In fact, a branch-specific model of evolution indicates that the estimated Ka/Ks ratio in the early stage of duplicate gene evolution is slightly lower than in the later stage (Table 1). When these data were partitioned by gene fragment the results were the same – there also was not a significant difference in the Ka/Ks ratio at the early compared to the later stage of duplicate gene evolution (Table 1). Additionally, a model in which the Ka/Ks ratio of the early lineage is allowed to be lower than one is significantly better than a model in which this rate ratio is fixed at the neutral expectation of one (P < 0.00001, Table 2) and this analysis also produced the same result when the data were partitioned by gene fragment (Table 2). Similarly, branch-site models recover a higher proportion of positively selected sites in the later lineage (0.00893%) than the early lineage (0.00061%; data not shown).

**Table 1:** Comparison of alternatively parameterized models of evolution in Fig. 1 indicates no significant difference in the Ka/Ks ratio at an early and a later stage of duplicate gene evolution.

Comparison	# base pairs	-lnL Ho	-lnL Ha	P value	Ka/Ks combined early and late	Ka/Ks ratio early	Ka/Ks ratio late	Ka/Ks diploid
Fig. 1B	80856	-165602.720	-165602.386	0.414	0.164	0.158	0.169	0.126
Fig. 1C	9717	-15699.366	-15697.250	1.000	0.208	0.124	0.346	0.198
Fig. 1D	6966	-13187.865	-13186.872	0.160	0.126	0.187	0.105	NA
Fig. 1B (partitioned)	80856	-160085.863	-159889.926	1.000	NL	Af2	Af2	NL
Fig. 1C (partitioned)	9717	-15400.349	-15393.089	0.888	NL	Af2	Af2	NL
Fig. 1D (partitioned)	6966	-12983.343	-12978.034	0.807	NL	Af2	Af2	NA

Indicated for comparisons depicted in Fig. 1B, C and D are likelihoods of the null model (early and later Ka/Ks are the same) and the alternative model (early and later Ka/Ks are not the same), the one-sided probability of the Ka/Ks ratio being higher in the early stage, and the Ka/Ks ratios estimated from each of these models. For the first two tests, the Ka/Ks ratio of the diploid lineage was estimated using a different model where a unique Ka/Ks ratio was estimated for each branch (a free ratio model). Also listed are the joint likelihoods of these models from an analysis partitioned by gene fragment. For the partitioned analyses, Ka/Ks ratios for each fragment are either listed in Additional file 2 (Af2), not listed (NL), or not applicable (NA).

**Table 2:** Comparison of alternatively parameterized models of evolution indicates significant departure from neutrality at an early stage of duplicate gene evolution.

Comparison	# bp	-lnL Ho	-lnL Ha	P value	Fixed Ka/Ks ratio in early lineage in null model	Estimated Ka/Ks ratio in other lineages in null model	Estimated Ka/Ks ratio in early lineage in alternative model	Estimated Ka/Ks ratio in other lineages in alternative model
Fig. 1B	80856	-166032.2641	-165608.1273	0.0000	1	0.1322	0.158	0.1415
Fig. 1C	9717	-15716.3195	-15698.0601	0.0000	1	0.2261	0.1242	0.228
Fig. 1D	6966	-13235.97398	-13187.03582	0.0000	1	0.141	0.1052	0.1557
Fig. 1B (partitioned)	80856	-160755.0615	-160071.0687	0.0000	1	NL	NL	NL
Fig. 1C (partitioned)	9717	-15436.44329	-15413.5068	0.0000	1	NL	NL	NL
Fig. 1D (partitioned)	6966	-13077.6404	-13016.5679	0.0000	1	NL	NL	NL

Likelihoods of a null model with the Ka/Ks ratio fixed at one at an early stage of duplicate gene evolution and an alternative model with this ratio estimated are indicated. Species acronyms are the same as in Fig. 1 and abbreviations are the same as in Table 1.

**Figure 2:**
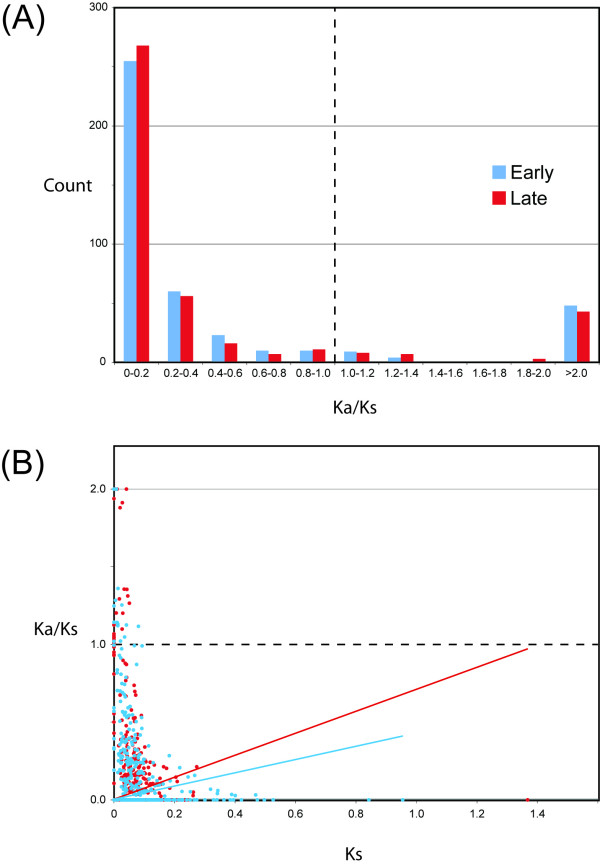
**Functional constraints are similar in early and later stages of duplicate gene evolution in *X. laevis *paralogs**. (A) Binned Ka/Ks of early (blue) and later (red) stages of duplicate gene evolution. (B) Regression of Ka/Ks versus Ks in the early and later stages indicates that selection (relaxed purifying + positive) is not more common in the early stage of duplicate gene evolution (blue dots) than the later stage (red dots). The Y-intercept of these regression lines was set to zero and Ka/Ks ratios greater 2 (including undefined ratios) were given a value of 2. In (A) and (B), a dashed line indicates the neutral expectation. Fragments with Ka/Ks > 2 are, on average, half of the size of those with Ka/Ks < 2. Ka/Ks ratios above 2 may therefore be attributable in part to stochastic variance in Ks [43].

Tests of the individual loci have low power because many are small fragments (see Additional file 2). Nonetheless, analyses of 660 fragments from 350 individual loci echo the results of the analyses of combined multi-locus data. The distribution of Ka/Ks ratios in the early and later stages of duplicate gene evolution is similar (Fig. 2A) and more fragments have a significantly higher Ka/Ks ratio at a later stage (8 fragments) than at an earlier stage (6 fragments), and this difference is not significant (χ^2 ^test, P = 0.997). Additionally, the number of fragments with a higher Ka/Ks ratio in the early stage than the later stage (significant or not) was lower (156 fragments) than the alternative (262 fragments; P = 1.0; see Additional file 2). That Ks in the early stage of duplicate gene evolution was similar to or lower than in the later stage (Fig. 1, see Additional file 3), indicates that sampling bias of synonymous substitutions [32,43], if present, would bias our analysis of individual fragments towards detecting a higher Ka/Ks ratio in the early stage, which is not what we observed.

The neutral expectation (Ka/Ks equal to one) is significantly rejected in the early lineage of 62 out of 136 individual loci with more than 200 bp (see Additional file 2), and when this ratio is estimated for the early lineage, only 7% of them have an estimated Ka/Ks ratio above one. Taken together, these results indicate that purifying selection was as strong, if not stronger on these duplicates in the early stage of their evolution compared to the later stage.

Early neofunctionalization could potentially result in no difference between the Ka/Ks ratio in the early and later stages of duplicate gene evolution if genes in the early stage experience either positive selection or purifying selection, whereas genes in the later stage experience either relaxed purifying selection or purifying selection. While we can not rule this possibility out because positive selection and relaxed purifying selection both increase the Ka/Ks ratio, a regression of Ka/Ks to Ks for each fragment in the early and later stage of evolution indicates that (positive + relaxed purifying) selection is less prevalent in the early stage than the later stage (Fig. 2B).

### Radical amino acid substitutions are not more common in early versus later stages of duplicate gene evolution

New function may be achieved by "radical" substitutions – replacement of one amino acid with another that has very different physical properties [24,44]. While this is certainly not a requirement for new function to evolve, we nonetheless explored this possibility using a Bayesian approach to estimate the number and frequencies of elemental substitutions – the 75 amino acid substitutions that can occur via a single nucleotide change – at an early and a later stage of duplicate gene evolution, and also in the diploid lineage (see Additional file 3). Results indicate that elemental substitutions were not more radical in an early stage (Mantel Z statistic = 2.4119) than in a later stage (P = 1.0000). In fact, radical substitutions were slightly more prevalent in the later lineage (Mantel Z statistic = 2.4680). Elemental substitutions also were not significantly more radical in the entire *X. laevis *paralog α lineage (between node 1 and XLα of Fig. 1B, Mantel Z statistic = 2.43823) than in the diploid lineage (between ST and node 1 in Fig. 1B; Mantel Z statistic = 2.3920, P = 0.1396). Similar results are obtained when the radicalness of elemental substitutions is categorized according to alternative criteria [data not shown; [45]].

Simulations were performed to test whether ancestral bias toward more conservative substitutions in the early stages of duplicate gene evolution could explain these results, but this was not the case. Simulated elemental substitutions from a reconstructed ancestral sequence were not more conservative in the early stage of duplicate gene evolution than the later stage (P = 0.6529). As expected, these simulations, which were not under purifying selection, were significantly more radical than the observed data (P < 0.001).

### Caveats

We performed additional analyses to address various concerns about the sequence dataset from *X. laevis*, *X. borealis*, and *S. tropicalis*. One consideration is that differences or changes in population size could affect the Ka/Ks ratio because slightly deleterious nonsynonymous substitutions are more likely to fix when the effective population size is small. Based on the geographic distribution and molecular diversity of mitochondrial DNA [39], the effective population size of *X. borealis *is smaller than that of *X. laevis*. However, we found that the Ka/Ks ratio of *X. laevis *paralogs during the later stage was slightly higher (0.1555) than the corresponding lineage of *X. borealis *(0.1338). This discrepancy was not significant in a two-sided test (P = 0.1790) or in a one-sided test because we expected the ratio to be larger in *X. borealis *(P = 1.0). To ensure that we were comparing ratios in expressed duplicates in both species, we included in this comparison only those data for which expression of both paralogs of both species was confirmed (37,194 bp). We note that more substitutions of both types occurred in *X. borealis *suggesting that the overall rate of evolution may be slightly higher in this species. A lack of significant difference in the Ka/Ks ratio suggests that the difference in effective population size between *X. laevis *and *X. borealis *had a negligible impact on the Ka/Ks ratios of many of their orthologs.

A second consideration stems from the possibility that a substantial portion of the early lineage of duplicate gene evolution evolved in a diploid (between nodes 1 and 2 in Fig. 1B) as a result of the putative allopolyploid origin of the ancestor of *Xenopus *tetraploids. Because the Ka/Ks ratio of clawed frog paralogs is slightly higher after genome duplication than before it [32,41], the Ka/Ks ratio of this entire branch (between nodes 1 and 3 in Fig. 1B) could be lower than the Ka/Ks ratio of the portion of this branch that evolved after duplication (between nodes 2 and 3 in Fig. 1B). To explore this issue, we analyzed expressed sequences from another dataset derived from *S. tropicalis *and two closely related tetraploids (9717 bp). Similar to the analysis of *X. laevis *and *X. borealis *paralogs, the branch-specific tests of *Silurana *paralogs do not provide evidence for an increased Ka/Ks ratio in an early stage (between nodes 7 and 8 in Fig. 1C) versus a later stage of duplicate gene evolution (between node 8 and EPα in Fig. 1C; P = 1.0; Table 1), nor an increased frequency of radical amino acid substitutions at an early stage of duplicate gene evolution (Mantel Z statistic = 2.7193) compared to a later stage (Mantel Z statistic = 2.1991, P = 0.0882). Simulations indicate that the early stage of duplicate gene evolution in *Silurana *was not significantly biased towards more conservative substitutions (P = 0.5651), the branch-site test recovers no evidence in the concatenated data for positively selected sites in the early branch (although it does on the later branch; data not shown), and the partitioned branch model analysis recovers the same results as the concatenated branch model (Tables 1 and 2). Also similar to the analysis of *X. laevis *and *X. borealis *paralogs, the branch-specific tests of *Silurana *paralogs illustrate that functional constraints during the early stage of duplicate gene evolution were significantly below neutral expectations (Table 2).

A third consideration is that allotetraploidization of the common ancestor of *Xenopus *tetraploids occurred immediately before the first speciation of this ancestor (in other words that the time between nodes 2 and 3 in Fig. 1B is very small). If this were the case, then it would be more informative to compare an "intermediate" stage of duplicate gene evolution – a period after the first tetraploid speciation in *Xenopus *but before subsequent tetraploid speciations (i.e. between nodes 3 and 4 in Fig. 1D) – to a later stage of duplicate gene evolution – after an even more recent tetraploid speciation event (between node 4 and XLα in Fig. 1D). This issue was addressed with additional sequences (6966 bp) from the tetraploid species *X. gilli *and *X. muelleri *that made possible the further dissection and hypothesis testing of the temporal dynamics of evolution after duplication (Fig. 1D). Based on their close phylogenetic relationships [22,39,46], we used *X. gilli *when we knew both *X. laevis *paralogs were expressed, and we used *X. muelleri *when we knew both *X. borealis *paralogs were expressed. Similar to the other analyses, this comparison revealed that the Ka/Ks ratio is not significantly higher in the intermediate stage compared to the later stage of duplicate gene evolution (P = 0.16; Table 1) and that the frequency of radical amino acid substitutions at the intermediate stage of duplicate gene evolution (Mantel Z statistic = 2.4073) is not significantly higher than at a later stage (Mantel Z statistic = 2.0645, P = 0.0887). Simulations again confirm that the intermediate stage was not significantly biased towards more conservative substitutions (P = 1.0000), the branch-site test recovers no evidence in the concatenated data for positively selected sites in the early branch or the later branch (data not shown), and the partitioned branch model analysis again recovers the same results as the concatenated branch model (Tables 1 and 2). These additional analyses thus provide strong support that purifying selection acted rapidly – within millions of years – and persistently – over tens of millions of years – after WGD in clawed frogs.

### Expression divergence

We used microarray data to compare expression profiles from five developmental stages and adult tissue types (treatments) of hundreds of paralogous pairs generated by WGD. Our analyses included developmental treatments from four distinct developmental stages (egg, tadpole stage 11, tadpole stage 18, and adult). Unlike the egg and tadpole stages, however, the adult stage is represented by data from each type of gonad instead of the entire individual. Because the primordial germ cells appear long after the tadpole stages that we assayed [stage [44,47]], these data provide a coarse perspective on spatial expression in four distinct tissue types: undifferentiated egg, pooled embryonic tissue (which do not have developed gonads), adult testis, and adult ovary.

We performed a power analysis to explore the possibility that cross-hybridization of non-target paralogs could affect the inference of paralogous expression profiles. We compared results from (a) a low paralog specificity analysis that included all probes on the microarray, including ones that cross hybridize to both paralogs, (b) a medium paralog specificity analysis that excluded probes whose sequences cross-hybridized to both paralogs, and (c) a high specificity analysis that excluded probes having up to three mismatches with a nontarget paralog. Additionally, we used two intensity thresholds, "standard" and "conservative", as a basis for the detection of expression of each paralog in each treatment (see Methods).

**Figure 3:**
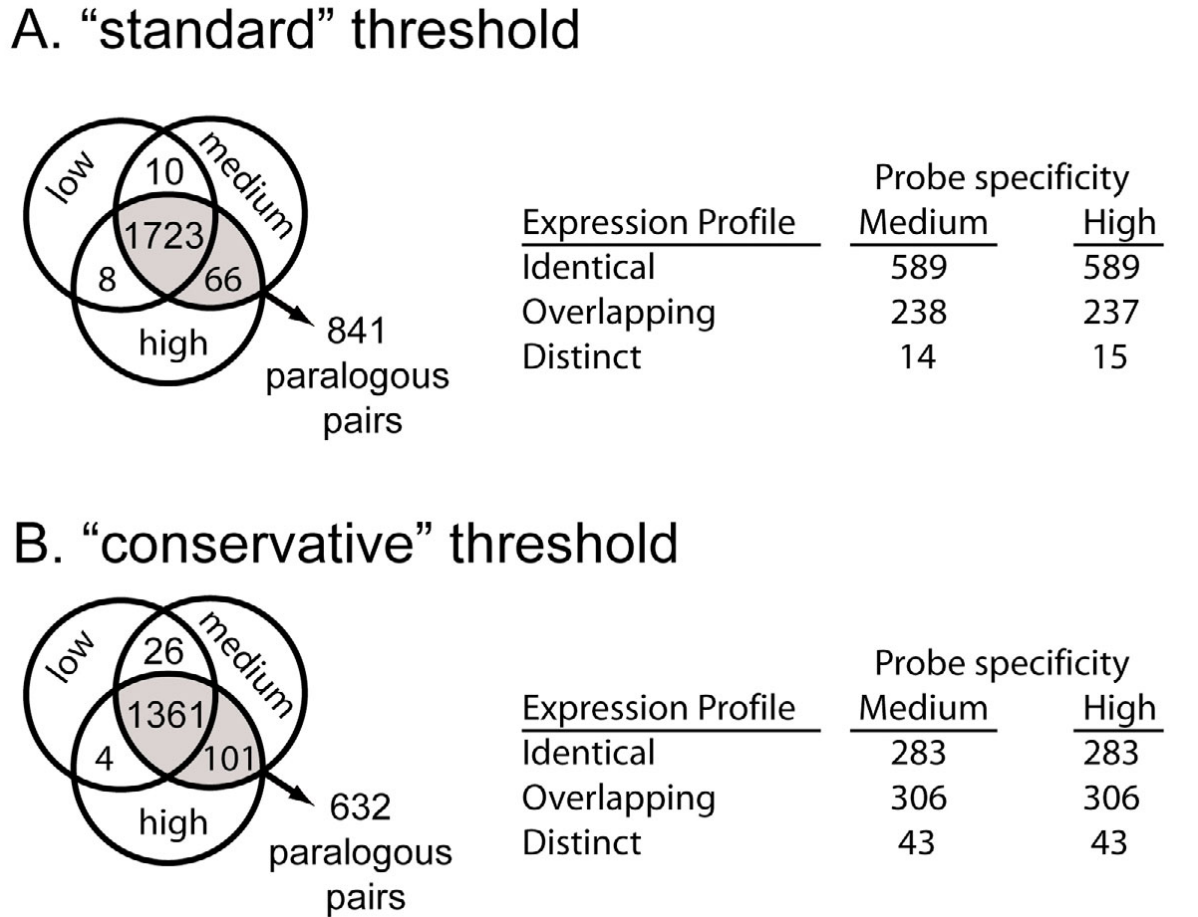
**Expression of both paralogs is generally detected in the same treatments, irrespective of the probe specificity (the degree to which each probe matches one but not the other paralog) or the detection threshold (the minimum raw intensity scored as expressed)**. These data are based on (A) "Standard" and (B) "Conservative" threshold levels for detection of expression and three probe specificities were compared that are labeled low, medium, and high (see Methods). We report paralogous profiles whose presence/absence scores in all five treatments were identical in the medium and high specificity analysis (shaded in gray on the left of each chart). 1789 and 1462 genes had consistent present/absent expression profiles in the medium and high specificity analyses using the standard and conservative thresholds. These sets of genes included 841 and 632 paralogous pairs, respectively. The tables on the right compare paralogous profiles by tabulating whether they are both present and absent in the same treatments (identical), the expression profile of one overlaps entirely with the other (overlap), or paralogs in which each duplicate has a unique component (distinct).

**Figure 4:**
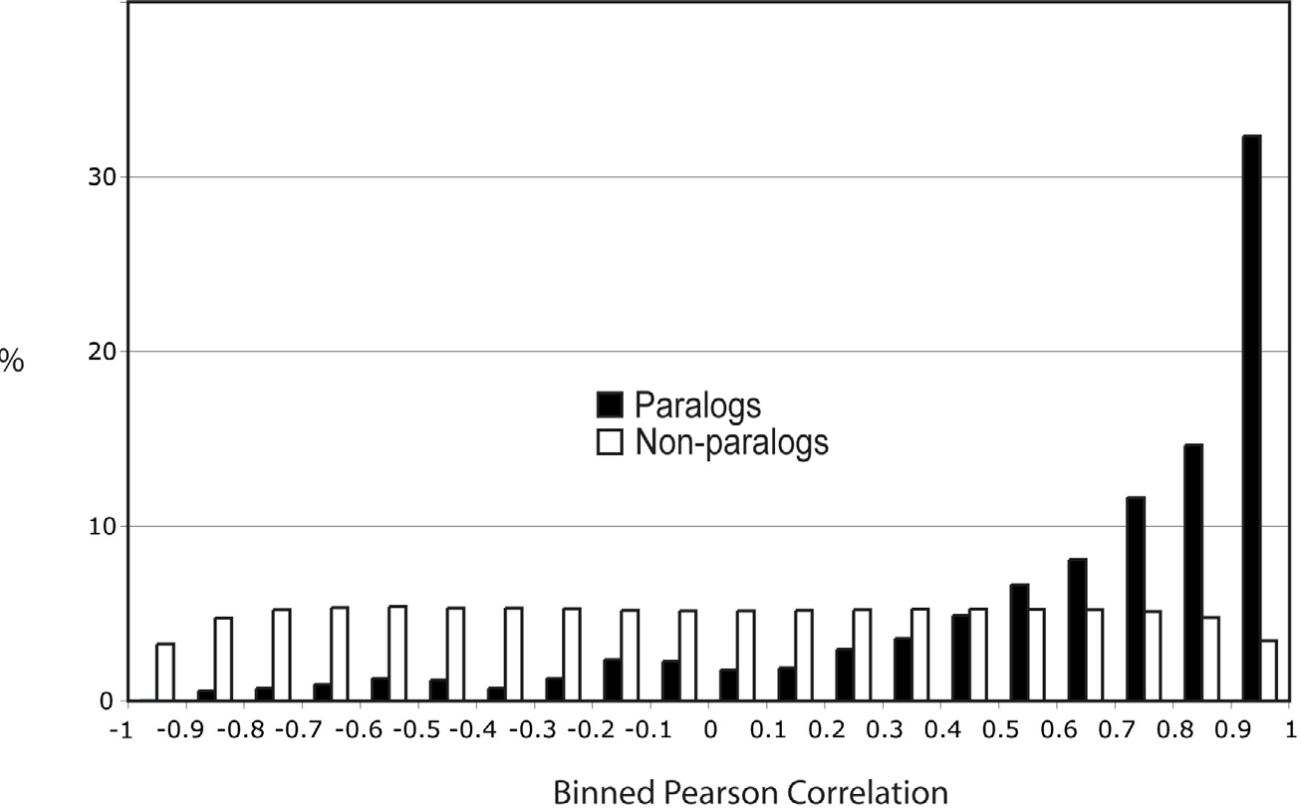
**Binned expression profile correlations between 841 pairs of paralogs over five developmental stages or adult tissue types in the medium specificity analysis**. The proportion of Pearson correlation coefficients between non-paralogous expression profiles (white bars) and between paralogous expression profiles (black bars). Ninety percent of the non-paralogous expression profiles have a Pearson correlation coefficient that is greater than -0.861 but less than 0.865. The Pearson correlation coefficients of 62% of the paralogous expression profiles are less than 0.865, and 0.3% of them are less than -0.861.

Qualitative comparisons across this developmental series and these tissue types indicate that the bulk of paralogous expression divergence after WGD in clawed frogs is on a quantitative rather than a temporal dimension (Figs. 3, 4). This would be expected if these paralogs were expressed in a highly specific manner in only one of the developmental stages or tissue types that we analyzed. However, many of these paralogs were expressed in multiple tissue types and multiple developmental stages. Consider for instance the 841 paralogous pairs for which the presence/absence expression profile of each paralog was identical in the medium and high paralog specificity analyses (Fig. 3). In the medium specificity analysis at the standard threshold, 94% of these paralogous pairs were both expressed in at least two treatments and 75% were both expressed in all five treatments.

When both paralogs are expressed, comparison of their expression profiles can indicate either that (a) both are expressed at the same developmental stages and tissue types (identical spatial and temporal expression), (b) the profile of one paralog is a subset of that of the other one (overlapping spatial and temporal expression), or (c) both paralogs have distinct components to their expression profiles (distinct spatial and temporal expression). In the microarray expression data from *X. laevis*, when expression of both paralogs was detected, almost all pairs had identical or overlapping expression profiles in terms of the developmental stages and tissue types in which expression was detected (Fig. 3). This was true regardless of how conservatively we scored presence/absence of expression or the specificity of the probes on the microarray. Only 2–7% of these pairs included paralogs that both had a unique expression profile wherein one paralog is expressed at a developmental stage or a tissue type where the other one is not expressed, and vice versa (Fig. 3).

In contrast to the overall similarity in the developmental timing and locations of paralogous expression, quantitative aspects of a high percentage of paralogous pairs have diverged substantially (Fig. 4). In the medium paralog specificity analysis for example, 62% of the paralogous pairs had a Pearson correlation coefficient that was below 0.866, a value below which were 95% of the correlation coefficients between non-paralogous genes. 27% of the paralogous pairs had a correlation coefficient below 0.5. Similar proportions were recovered in the high paralog specificity analysis (results not shown). At the end of this extreme, 0.3% of the paralogous pairs (3 pairs) in the medium paralog specificity analysis had a correlation coefficient that was more negative than -0.861, a level below which were only 5% of the correlation coefficients of the non-paralogous expression profiles. These three paralogous pairs are expressed in all treatments according to the standard detection threshold and have the following accession numbers (NM_001092603 and NM_001091285, NM_001091759 and NM_001093475, and NM_001091931 and NM_001094047). Their annotations are rudimentary, but the first pair may be involved with RNA splicing and the third pair has sequence similarity to collagen alpha(1) precursor. The normalized expression level of each pair indicates that in most of these treatments, the expression of one paralog is above the median expression level of that paralog across the five treatments whereas the expression of the other paralog is below it.

## Discussion

Neutral evolution of gene duplicates eventually leads to pseudogenization of one copy, and the time for this to occur depends on the size of the mutational target (sequence and length of the gene and the level of degeneracy of *cis*-regulatory elements), the rate and biases of molecular evolution (such as the rates of nucleotide substitution, insertions/deletions, and transposable element mobility), and the effective population size of the species (pseudogenes take longer to fix in larger populations) [48-50]. Non-neutral evolution, however, can curtail pseudogenization. In polyploid clawed frogs, duplicates generated by WGD are subject to more severe functional constraints than the neutral expectation, even though these constraints are relaxed relative to a singleton gene [this study; [32,40-42]]. Furthermore, even though the typical half-life of duplicates from a variety of organisms [6] is much lower than the time since tetraploidization of clawed frogs, it is clear that many paralogous pairs are still expressed in *Xenopus *[32,40-42], suggesting the action of natural selection to preserve their expression. If these paralogs are retained for enough time, functional constraints presumably would increase to a pre-WGD level. However, here we demonstrate that these constraints did not substantially fluctuate for dozens of millions of years following genome duplication.

One explanation for this observation is that the early stages of duplicate gene evolution occurred before these genomes became disomic (diploidized), and that this resulted in increased purifying selection on both duplicates in the early stages of their evolution. Indeed, some chromosomes may take longer than others to evolve disomic inheritance after WGD [3,51,52] and polysomic inheritance has been reported at one locus in the dodecaploid species *X. ruwenzoriensis *[53]. However, we removed from our analysis sequences that exhibited signs of gene conversion or recombination (see Additional file 3) – events that might indicate polysomic rather than disomic inheritance. Additionally, disomic inheritance can occur instantly or soon after WGD by allopolyploidization [54] and disomic inheritance of alleles occurs immediately in laboratory generated polyploids of *Xenopus *[55]. These observations argue against functional constraints on these paralogs being buoyed by polysomic inheritance in an early stage after allotetraploidization.

The stasis of functional constraints over these early stages of paralog evolution in clawed frogs contrasts sharply with studies of young and older duplicates generated from WGD in non-vertebrates and from segmental duplication in vertebrates. For example, over a level of synonymous divergence similar to *Xenopus *paralogs, older paralogs of the fungus and plant polyploids *Saccharomyces cerevisae *and *Arabidopsis thaliana *are more constrained than younger ones [6]. Likewise, human paralogs with synonymous divergence between 0.05 and 0.1 have a Ka/Ks ratio of about 0.47 but those with synonymous divergence between 0.1 and 0.5 are more constrained with a Ka/Ks ratio of about 0.37 [35]. Although those comparisons involve different sets of genes in each taxon, it is worth noting that functional constraints immediately after WGD are more severe in *Xenopus *paralogs, which have a lower Ka/Ks ratio of 0.105 – 0.158 (Table 1). These results suggest that (a) the evolutionary trajectories of duplicates generated by segmental duplication differ from those of paralogs generated by WGD and/or that (b) the early stage of evolution of duplicates that are destined to persist differs substantially from that of most young duplicates (the bulk of which rapidly degenerate to singletons). These results are consistent with the observation that young paralogs that evolve quickly are less likely to be retained in the long run [35,56-58]. Stoichiometric constraints/genic balance is one plausible explanation for more severe and persistent functional constraints on WGD paralogs in clawed frogs as compared to singletons in other organisms [6-8].

Temporal dynamics of molecular evolution of expressed duplicates appear to differ in frogs (this study) and yeast [59]. While purifying selection is relaxed after WGD in yeast and in *X. laevis*, nonsynonymous substitutions were more prevalent during an early stage of duplicate gene evolution than a later stage in yeast [59] but not in *X. laevis *(this study). There are multiple possible explanations for this difference. Because the yeast species examined in [59] have a larger effective population size than the frogs we studied, purifying selection in frogs would have to be stronger in order to substantially curtail the fixation of slightly deleterious nonsynonymous substitutions by genetic drift. Perhaps then, the initial phase of duplicate gene evolution – a period during which purifying selection is relaxed compared to singletons but before post-WGD increases in functional constraints are apparent at a molecular level – is more drawn out in frogs than in yeast as a consequence of their different population sizes. Another possibility is that the selective regime following WGD varies between yeast and frogs as a result of fundamental differences in the nexus of protein-protein interactions, functional specialization, complexity, and/or redundancy. It is also possible that the periods of time after WGD that were compared in each of these studies could differ substantially.

If post-duplication neofunctionalization of protein structure is to promote the persistence of both paralogs, amino acid changing nucleotide substitutions must occur in at least one paralog soon after duplication, and this should be followed by increased purifying selection once new function is acquired [1,60]. Molecular signs of neofunctionalization of protein structure may include a higher Ka/Ks ratio in early than in later stages of duplicate gene evolution, a higher frequency of radical amino acid changes in early than in later stages of duplicate gene evolution, and/or significantly different rates of nonsynonymous substitution between paralogs. In clawed frogs, multiple lines of evidence suggest that this mechanism is not a prevalent trigger for the persistence of duplicates generated in the initial millions of years after WGD. First, in the early stage of duplicate gene evolution in *X. laevis *only a handful of these persistent paralogs have a Ka/Ks ratio greater than one (i.e. consistent with positive selection; see Additional file 2) and a higher proportion of sites exhibit evidence of positive selection in the later stage of duplicate gene evolution than in the early stage (data not shown). Of course, the Ka/Ks ratio is a very rough metric of positive selection and new protein function could arise by neutral evolution, even via very few amino acid substitutions [61]. However, similar to yeast [59], radical amino acid substitutions are not more prevalent in the early stage of duplicate gene evolution. We also did not observe increased purifying selection in the later stage of duplicate gene evolution that would be expected if neofunctionalization occurred in the early stage after WGD. Similarly in yeast, duplicates with a level of divergence similar to *X. laevis *paralogs (Ks < 0.25), subfunctionalization as opposed to neofunctionalization is suggested by a loss of shared interactions [28,62].

These analyses recover a much higher incidence of quantitative divergence than the 14% suggested by [41], but they are similar to another study that suggests 40–50% quantitative expression divergence [42]. Hellsten et al. [42] found evidence of spatial expression divergence in four out of six *in situ *hybridizations, whereas we recovered this type of expression divergence – where each paralog has a unique component to its expression domain – in only 2–7% of the paralogs (Fig. 3). This disparity is in part a consequence of lack of resolution in the microarray data that we analyzed relative to *in situ *hybridization performed by [42]. Spatial and temporal expression subfunctionalization may be more common on a finer spatial or temporal scale than we were able to detect with these microarray data.

Unequal expression and low correlation of paralogous expression profiles has also been reported in several allopolyploid plants [63,64]. Genome duplication in plants is associated with non-additive changes in gene expression, suggesting that expression divergence between paralogs can immediately accompany allopolyploidization [65-67]. In synthetic allopolyploid *Arabidopsis*, for example, expression of over 5% of genes in synthetic allopolyploid lines deviated from the midpoint of each parental species [65]. In the recently formed allohexaploid plant species *Senecio cambrensis*, expression analysis of re-synthesized lines suggests that the impact of hybridization and genome duplication on expression divergence are distinct, and that the latter phenomenon can reduce expression divergence, at least in the early stages of polyploid evolution [68]. Later on, for example in *Arabidopsis thaliana *which experienced WGD between 20 and 60 million years ago, 57% of the resulting duplicates have an expression profile with a correlation coefficient less than 0.52 [64]. Likewise in yeast the correlation between paralogous expression profiles is lower than 0.5 in 55% of pairs that have a similar level of synonymous divergence (0.1–0.3) as the *X. laevis *paralogs in this study [69]. Substantial quantitative expression divergence between paralogs soon after WGD therefore does not appear to be unique to *X. laevis*, and is likely the culmination of divergence over evolutionary time and also divergence that occurred immediately upon allopolyploidization.

Without additional information on expression profiles of orthologous genes, at this point we cannot determine whether the observed spatial and temporal expression divergence arose through expansion (expression neofunctionalization) or degradation (expression subfunctionalization) of each expression profile or both. In yeast, expression neofunctionalization occurs via recruitment of *cis-*regulatory elements, but this appears to take a long time [70]. In human paralogs that are more diverged (Ks > 0.25) than the ones we studied here, the combined expression domains of segmental duplicates is typically larger than that of singletons, and the magnitude of this difference is positively correlated with synonymous divergence, suggesting expression neofunctionalization [28]. Expression divergence is correlated with synonymous and nonsynonymous divergence in yeast duplicates with Ka ≤ 0.3 or Ks < 1.5 [69], and this correlation has also been recovered in humans over similar levels of divergence [71]. However, we did not find this correlation in *X. laevis *paralogs (see Additional file 4). This difference could derive from distinct genetic fates of duplicates generated by WGD versus segmental duplication on either an expression or functional level [72]. Other factors that could play a role in the degree to which paralogous expression profiles diverge over time include tissue-specific developmental constraints [73], expression intensity and specificity [74], and the essentiality of a paralog's gene family [58].

## Conclusion

It has been suggested that allopolyploidization rather than autopolyploidization preceded the diversification of jawed vertebrates [75]. Allopolyploids have the advantage that diploidization might occur instantly or more rapidly than in autopolyploids, thereby preventing complications associated with mis-segregation of chromosomes in a polysomic genome [54]. By analogy, duplicate gene evolution in allopolyploid clawed frogs offers insights into how the transcriptome of our ancient ancestors may have been sculpted in the wake of these genomic metamorphoses [1,37], and also after subsequent WGDs in other vertebrates [2,76]. To the extent that this analogy applies, the initial dozens of millions of vertebrate evolution after WGD were likely characterized by strong and persistent functional constraints at the amino acid level. Despite these functional constraints, however, quantitative expression divergence probably occurred in many duplicates during this period and, as has been suggested [3], the magnitude of regulatory and structural change was not correlated (see Additional file 4). We speculate therefore that stoichiometric requirements and quantitative expression subfunctionalization commonly trigger persistence of WGD paralogs in the earliest stages of their existence. Following WGD, it appears that other mechanisms that trigger the retention of duplicate genes, such as neofunctionalization of the coding region or spatial expression subfunctionalization [e. g. [25,28,77-79]], tend to operate less frequently, later, or over a longer period of time. Interestingly, analysis of teleost paralogs demonstrates that duplicates continue to be lost over hundreds of millions of years [78], indicating that the steadfast functional constraints and substantial expression dynamics soon after vertebrate WGD do not immortalize these duplicates.

## Methods

### Molecular data

We compiled sequences of expressed paralogs of *X. laevis *from Genbank and various publications[32,41,42] and aligned them with orthologs from the *S. tropicalis *genome assembly 4.1. 454 pyrosequencing was used to obtain sequences of fragments of expressed paralogs of *X. borealis *from testis cDNA and contigs were assembled from these data using BLAST [80] and ALIGN0 [81] from the FASTA 2.0 package [82], and manual alignment in MacClade [83]. Manufacturer protocols were followed to isolate RNA using an RNA extraction kit (Qiagen), to prepare cDNA using BD SMART PCR cDNA synthesis kit (Clontech), and to normalize the cDNA using the Trimmer cDNA normalization kit (Evrogen JCS). Additional targeted sequencing of paralogs from *X. laevis*, *X. borealis*, *X. gilli*, *X. muelleri*, *S. epitropicalis*, and *S*. new tetraploid was performed by co-amplifying portions of these paralogs from cDNA from a variety of tissues (blood, heart, brain, testis, liver, muscle). Portions of individual paralogs were then cloned with the TA cloning kit (Invitrogen) and sequenced. These data are deposited in Genbank (see Additional file 2).

Using a combination of targeted amplification, cloning, and sequencing of cDNA, 454 pyrosequencing of cDNA, and database searches, 80,856 bp were collected from 660 fragments of 350 expressed paralogous pairs from the tetraploid *X. laevis*, one expressed paralog from the tetraploid *X. borealis*, and an ortholog from the diploid *S. tropicalis*. An additional 9,717 bp were sequenced from portions of thirteen expressed duplicated loci of the tetraploids *S. epitropicalis *and *S*. new tetraploid, and 6,966 bp were sequenced from portions of nine expressed duplicated loci of the tetraploids *X. muelleri *or *X. gilli*. To minimize analysis of paralogs whose evolutionary history may have been homogenized by gene conversion or recombination, we excluded from our analysis sequences with signs of these phenomena (see Additional file 1).

Because data were usually obtained from only one expressed *X. borealis *paralog but two *X. laevis *paralogs, most of our molecular analyses focused on molecular evolution of one *X. laevis *paralog – the "α" paralog (Fig. 1B). This is because, without evidence of expression of the other *X. borealis *paralog, we do not know whether *X. borealis *paralog α is still an expressed duplicate, and we also cannot determine at what point after duplication nonsyonymous substitutions occurred in the other *X. laevis *paralog – paralog "β" (Fig. 1B). Phylogenetic methods (maximum parsimony, maximum likelihood) were used to identify to which one of the expressed *X. laevis *paralogs that the *X. borealis *paralog was most closely related.

### Models of evolution

To test whether the rate ratio of nonsynonymous to synonymous substitutions per site (hereafter the Ka/Ks ratio) differs at early versus later stages of duplicate gene evolution, the likelihood of alternative models of branch-specific evolution (Fig. 1) was calculated using PAML version 3.15 [84]. This analysis was performed on concatenated datasets and with the data partitioned by gene fragment. We also used the branch-site test for positive selection [test [2] in [85]] to test whether there were more sites under positive selection at an early stage compared to a later stage of duplicate gene evolution. In addition, we tested for significant departure from neutrality by comparing a model in which the Ka/Ks ratio of the early lineage was fixed at one and another Ka/Ks ratio was estimated for all other branches, to a model in which one Ka/Ks ratio was estimated for the early branch and another ratio was again estimated for all other branches. For each comparison, significance of the more parameterized model was evaluated with a χ^2 ^test. Note that, as a result of a suspected allotetraploid origin of the ancestor of *X. laevis *and *X. borealis*, an unknown portion of the early lineage probably evolved in a diploid species; the potential impact of this and other caveats was explored with additional comparative data and analyses (Figs. 1C, D).

### Expression analyses

We collected expression data from previous studies that used a *X. laevis *microarray prefabricated by Affymetrix [86-88]. Expression data was analyzed from five developmental stages or tissue types: egg, embryonic stages 11 and 18, adult testis, and adult ovary. Raw intensity data were converted to CEL files using GeneChip Operation System software (GCOS v. 1.4 Affymetrix). The robust multi array average (RMA) algorithm was implemented to quantify gene expression in GeneSpring version GX7.3 (Agilent, Inc) using either the Affymetrix library file or custom CDF files ("probe masks") that were generated following Hammond et al. [89]. The data were then normalized to the median of each gene across all arrays and the 50th percentile of each array. A high intra-treatment correlation (R^2 ^= 92–98%) was found between the biological replicates for each treatment.

The Affymetrix *X. laevis *microarray consists of "probe sets" that are composed of 16 "probe pairs", each of which includes a 25 base pair oligo that is intended to perfectly match the target sequence. Cross hybridization of paralogs could homogenize their expression profiles if it is bidirectional or could amplify differences between them if it is unidirectional. To explore this possibility, we performed a power analysis in which we used probe masks to evaluate paralog specificity of each probe set – i.e. the degree to which the probes on the microarray match one paralog but not the other. We tested three paralog specificities: "low", "medium", and "high". The low paralog specificity analysis included probes that exactly matched (and cross-hybridize to) both paralogs. The medium paralog specificity analysis excluded probes that exactly matched both paralogs. The high paralog specificity analysis excluded probes that perfectly matched both paralogs and also those that had up to and including three mismatches with the non-target paralog. We required each probe set in our analysis to have a minimum of at least 8 probe pairs (and up to 16) at the highest specificity. These probe masks were developed based on comparisons of the probe sequences to a sequences of expressed paralog pairs from previous publications [32,41,42] that were carried out using BLAST searches [80]. We evaluated each of these probe specificities under two thresholds for calling presence/absence of expression ("standard" and "conservative" thresholds; Fig. 3). For the "standard" threshold, a paralog was scored as expressed if its raw intensity was above a background level of 50. For the "conservative" threshold, a paralog was scored as expressed if its raw intensity was above a background level of 200. We note that these thresholds are somewhat arbitrary because some probesets may hybridize with lower affinities than others, and therefore recover lower than background raw intensities even though a transcript is in fact expressed. This approach therefore provides only a rough metric of whether or not a transcript is expressed. The Pearson correlation coefficients provide an alternative extreme, because they are based on the expression intensities in all treatments (even those that have below-background raw intensities). These correlation coefficients therefore must be interpreted with the caveat that higher correlations between tissue profiles could be obtained when neither transcript is expressed in many treatments. To contextualize the paralogous correlations, we also calculated the Pearson correlation coefficients between all non-parlaogous expression profiles as in [64].

## Authors' contributions

FJJC and BJE conceived of the project and performed the molecular analyses. FJJC and DI performed the microarray analyses. FJJC and BJE wrote the manuscript and FJJC, BJE, and DI edited the manuscript. All authors have read and approved the final manuscript.

## Supplementary Material

Additional file 1 **Binned rates of synonymous substitution per site (Ks) of paralog α of gene fragments greater than 200 bp suggest that Ks is lower in the early stage than in the later stage**. Ks values were calculated using a free ratio model on the phylogeny depicted in Fig. 1B in which Ks is estimated independently for each branch. The early stage of evolution (blue bars) corresponds with the paralog α lineage between node 1 and 3 and the later stage of evolution (red bars) corresponds with the XLα lineage between node 3 and XLα. Click here for file

Additional file 2 **Information about sequence data including gene acronym, length in base pairs (bp), and Genbank accession numbers, and results of model based analysis of individual fragments**. Gene acronyms refer to the name of one *Xenopus *paralog or, if a name was not available, an acronym of a closely related named homolog. *Xenopus borealis *sequences less than 50 bp in length were not submitted to Genbank and are available upon request (AUR). Species and paralog abbreviations are the same as in Fig. 1. Discontinuous fragments of the same paralog have separate accession numbers. For each fragment, the likelihood of a null (Ho) and alternative (Ha) model of evolution is listed for two tests that correspond with the combined analyses presented in Tables 1 and 2. If the P value is greater than 0.05 the null model is not rejected. For the first test, in which the alternative model has a different Ka/Ks ratio in the early and later stages of duplicate gene evolution, the estimated Ka/Ks ratios are listed. Note that the null model of no difference between these ratios is not rejected for most fragments. Click here for file

Additional file 3 supplementary methods. Click here for file

Additional file 4 **No correlation between expression divergence and (A) Ka, (B) Ks, or (C) Ka/Ks (R^2 ^≅ 0.0002 and P > 0.50 for all correlations)**. Expression divergence is quantified by ln(1+R)/(1-R) where R is the Pearson correlation coefficient between each paralogous expression profile [69]. In (C) two outliers that have a Ka/Ks ratio over 1 are excluded. There also is not a significant correlation between the Ka/Ks ratio and ln(1+R)/(1-R) (data not shown). Ka/Ks ratios were calculated from complete or large fragments of expressed *X. laevis *paralogs; the average length of these sequences was 1119 bp. Click here for file
