# Supplementary figures and images for: Duplicate gene evolution and expression in the wake of vertebrate allopolyploidization
Source: BMC Evol Biol. 2008 Feb 8;8:43. doi: 10.1186/1471-2148-8-43 (PMC2275784; doi:10.1186/1471-2148-8-43)

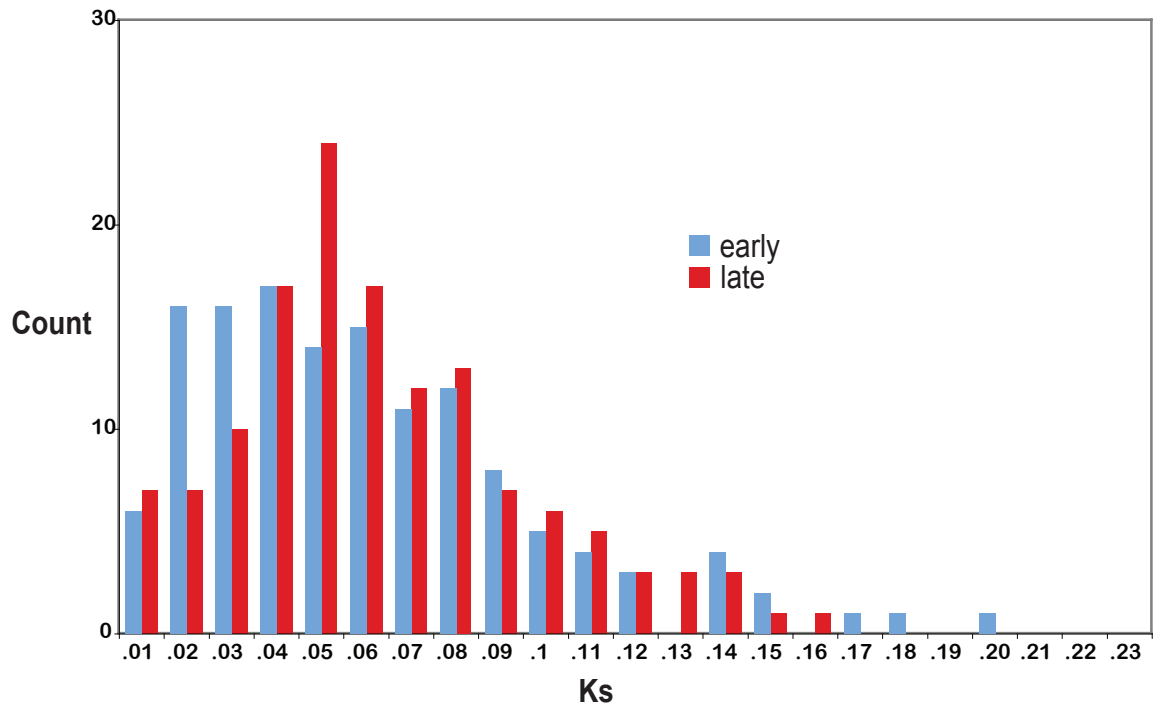

Supplement: Additional file 1 — Binned rates of synonymous substitution per site (Ks) of paralog α of gene fragments greater than 200 bp suggest that Ks is lower in the early stage than in the later stage. Ks values were calculated using a free ratio model on the phylogeny depicted in Fig. 1B in which Ks is estimated independently for each branch. The early stage of evolution (blue bars) corresponds with the paralog α lineage between node 1 and 3 and the later stage of evolution (red bars) corresponds with the XLα lineage between node 3 and XLα. [file 1471-2148-8-43-S1.PDF]

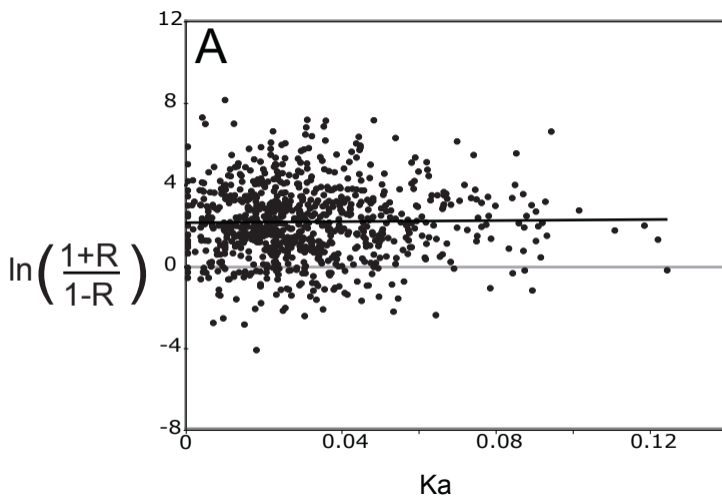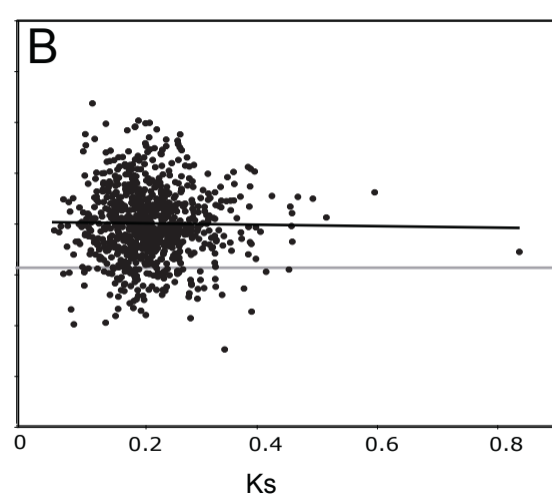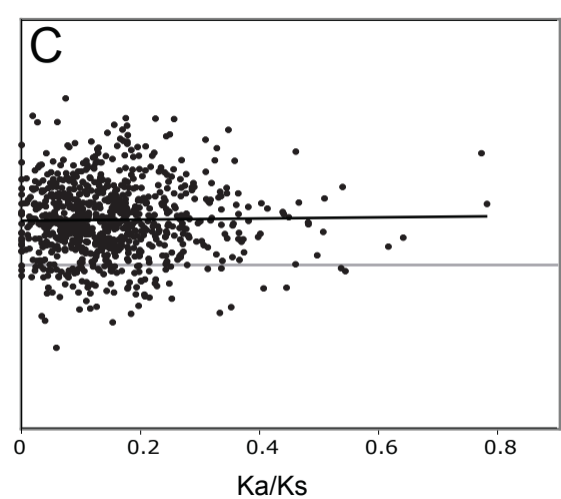

Supplement: Additional file 4 — No correlation between expression divergence and (A) Ka, (B) Ks, or (C) Ka/Ks (R2 ≅ 0.0002 and P > 0.50 for all correlations). Expression divergence is quantified by ln(1+R)/(1-R) where R is the Pearson correlation coefficient between each paralogous expression profile [69]. In (C) two outliers that have a Ka/Ks ratio over 1 are excluded. There also is not a significant correlation between the Ka/Ks ratio and ln(1+R)/(1-R) (data not shown). Ka/Ks ratios were calculated from complete or large fragments of expressed X. laevis paralogs; the average length of these sequences was 1119 bp. [file 1471-2148-8-43-S4.PDF]
