# Supplementary material for: Duplicate gene evolution and expression in the wake of vertebrate allopolyploidization
Source: BMC Evol Biol. 2008 Feb 8;8:43. doi: 10.1186/1471-2148-8-43 (PMC2275784; doi:10.1186/1471-2148-8-43)
Supplement: Additional file 2 — Information about sequence data including gene acronym, length in base pairs (bp), and Genbank accession numbers, and results of model based analysis of individual fragments. Gene acronyms refer to the name of one Xenopus paralog or, if a name was not available, an acronym of a closely related named homolog. Xenopus borealis sequences less than 50 bp in length were not submitted to Genbank and are available upon request (AUR). Species and paralog abbreviations are the same as in Fig. 1. Discontinuous fragments of the same paralog have separate accession numbers. For each fragment, the likelihood of a null (Ho) and alternative (Ha) model of evolution is listed for two tests that correspond with the combined analyses presented in Tables 1 and 2. If the P value is greater than 0.05 the null model is not rejected. For the first test, in which the alternative model has a different Ka/Ks ratio in the early and later stages of duplicate gene evolution, the estimated Ka/Ks ratios are listed. Note that the null model of no difference between these ratios is not rejected for most fragments. [file 1471-2148-8-43-S2.DOC]

**Additional file 2 - Information about sequence data including gene acronym, length in base pairs (bp), and Genbank accession numbers, and results of model based analysis of individual fragments.**

Gene acronyms refer to the name of one *Xenopus* paralog or, if a name was not available, an acronym of a closely related named homolog. *Xenopus borealis* sequences less than 50 bp in length were not submitted to Genbank and are available upon request (AUR). Species and paralog abbreviations are the same as in Fig. 1. Discontinuous fragments of the same paralog have separate accession numbers. For each fragment, the likelihood of a null (Ho) and alternative (Ha) model of evolution is listed for two tests that correspond with the combined analyses presented in Tables 1 and 2. If the P value is greater than 0.05 the null model is not rejected. For the first test, in which the alternative model has a different Ka/Ks ratio in the early and later stages of duplicate gene evolution, the estimated Ka/Ks ratios are listed. Note that the null model of no difference between these ratios is not rejected for most fragments.
