# Supplementary material for: Duplicate gene evolution and expression in the wake of vertebrate allopolyploidization
Source: BMC Evol Biol. 2008 Feb 8;8:43. doi: 10.1186/1471-2148-8-43 (PMC2275784; doi:10.1186/1471-2148-8-43)
Supplement: Additional file 3 — supplementary methods. [file 1471-2148-8-43-S3.DOC]

**Additional File 3: Supplemental methods**

**Tests for recombination and gene conversion.**  To explore the possibility that recombination or gene conversion occurred among these paralogs, multiple tests were used because their performance varies with the level of divergence, the extent of recombination, and among site rate heterogeneity [1, 2]. Tests for recombination include the recombination detection program, geneconv, chimera, bootscan, and siscan, as implemented by the Recombination Detection Program [2-7]. A variety of parameter settings were explored for each method as in [8], and only paralogs with more than 300 bp were analyzed using these tests.

A site is parsimony-informative if it contains at least two types of nucleotides that each occur in at least two taxa. Thus, when analyzing phylogenetic relationships among four taxa, the only character pattern that is parsimony-informative is one in which two taxa share one nucleotide and the other two share a different nucleotide. Using this principal, we tabulated the number and order of parsimony-informative “non-recombined” character patterns, in which the α paralogs of *X. laevis* and *X. borealis* both had the same nucleotide and the β paralog of *X. laevis* and the ortholog of *S. tropicalis* both had a different nucleotide. Additionally, we tabulated the number and order of parsimony-informative “recombined” character patterns in which the α paralog of *X. laevis* and *X. borealis* each had a different nucleotide, but where each one was identical to the homologous nucleotide of the β paralog of *X. laevis* or the ortholog of *S. tropicalis*. Loci that had three or more consecutive “recombined” character patterns (which could derive from recombination or gene conversion between alleles of different paralogs), were excluded from our analysis. In one gene (Xmegs), a run of four recombined character patterns turned out to be a combination that included two nonrecombined character patterns when a fifth paralog was considered (*X. borealis* paralog β), so this locus was retained.

**Conservative versus radical changes after duplication.** We used a Bayesian approach to estimate the number and frequency of each of the 75 elementary amino acid changes at different time points after genome duplication. This approach employed a simulation procedure to stochastically map mutations on a fixed topology [9]. We attempted to accommodate uncertainty in branch lengths and parameter values by sampling 100 sets from a post-burnin posterior distribution that was generated from Bayesian analysis with a constrained topology using MrBayes version 3.1.2 [10]. This sample was used to simulate character evolution conditioning on the observed data and allowing all possible character states for each ancestral node with sampling of these states drawn according to their likelihood [9]. Simulations were performed using SIMMAP version 1.0 [11] and PERL scripts were used to reconstruct and tabulate each of the simulated elemental amino acid changes along each branch. Results were similar to those obtained from maximum likelihood analysis of amino acid substitutions.

A lineage with many radical amino acid substitutions has a low correlation between the frequency of each type of substitution and the magnitude of the biochemical differences between the ancestral and descendant amino acid residues. Mantel tests were used to calculate the correlation between the number of each type of elementary amino acid change and the associated biochemical transition associated with each substitution, based on eight physical properties [12]. To test whether this correlation was significantly different in the early stage of duplicate gene evolution than in a later stage, the Mantel Z statistic [13] from the early stage was compared to a distribution of Mantel Z statistics generated from 100,000 bootstrapped datasets derived from *n* draws from the multinomial frequency distribution estimated for the later stage, where *n* is a maximum likelihood estimate of the number of observed elemental substitutions in the early stage.

Simulations were performed to test whether phylogenetic inertia (an ancestral bias towards more or less conservative substitutions) could account for the observed proportion of radical and conservative substitutions at each stage of duplicate gene evolution. A maximum likelihood estimate of the ancestral sequence of nodes 1 and 3 in Fig 1A, nodes 2 and 3 in Fig. 1B, and nodes (23) and 4 in Fig. 1C, was obtained using PAML. For each branch, 100,000 simulations were performed from these ancestral sequences under the general time reversible model of evolution with a proportion of invariant sites and a gamma distributed rate heterogeneity parameter, using SeqGen version 1.3.2 [14]. The posterior sample of 100 sets of parameter values and corresponding branchlengths that were used in the stochastic mapping of mutations in the observed data were also used in these simulations. Simulated elemental substitutions were then inferred by maximum likelihood and maximum parsimony. Additionally, the PSEUDOGENE program was used to obtain a rough estimate expected half lives of these loci under neutral evolution, using values for the rate of point mutations and the rate of insertions and deletions estimated from old world primates, as in Zhang and Webb [15]. A reconstruction of the ancestral sequence of these paralogs was used for the simulations and the half-life was estimated for only those loci for which complete transcripts were available in both *X. laevis* paralogs.

**LITERATURE CITED**

1. Posada D: **Evaluation of methods for detecting recombination from DNA sequences: empirical data**. *Mol Biol Evol* 2002, **19**(5):708-717.

2. Posada D, Crandall KA: **Evaluation of methods for detecting recombination from DNA sequences: computer simulations.** *Proc Nat Acad Sci* 2001, **98**(24):13757-13762.

3. Gibbs MJ, Armstrong JS, Gibbs AJ: **Sister-Scanning: a Monte Carlo procedure for assessing signals in recombinant sequences.** *Bioinformatics* 2000, **16**:573-582.

4. Martin D, Rybicki E: **RDP: detection of recombination amongst aligned sequences**. *Bioinformatics* 2000, **16**(6):562-563.

5. Maynard Smith J: **Analyzing the mosaic structure of genes**. *J Mol Evol* 1992, **34**:126-129.

6. Padidam M, Sawyer S, Fauquet CM: **Possible emergence of new geminiviruses by frequent recombination**. *Virology* 1999, **265**:218-225.

7. Salminen MO, Carr JK, Burke DS, McCutchan FE: **Identification of breakpoints in intergenotypic recombinants of HIV-1 by bootscanning**. *AIDS Res Hum Retroviruses* 1995, **11**:1423-1425.

8. Evans BJ, Kelley DB, Melnick DJ, Cannatella DC: **Evolution of RAG-1 in polyploid clawed frogs**. *Mol Biol Evol* 2005, **22**(5):1193-1207.

9. Nielsen R: **Mapping mutations on phylogenies**. *Syst Biol* 2002, **51**(5):729-739.

10. Huelsenbeck JP, Ronquist F: **MrBayes: Bayesian inference of phylogenetic trees**. *Bioinformatics* 2001, **17**(8):754-755.

11. Bollback JP: **SIMMAP: stochastic character mapping of discrete traits on phylogenies**. *BMC Bioinformatics* 2006, **7**:88.

12. Urbina D, Tang B, Higgs PG: **The response of amino acid frequencies to directional mutation pressure in mitochondrial genome sequences is related to the physical properties of the amino acids and to the structure of the genetic code.** *J Mol Evol* 2006, **62**:340-361.

13. Sokal RR, Rohlf FJ: **Biometry**, Third Edition edn. New York: W. H. Freeman and Company; 2003.

14. Rambaut A, Grassly NC: **Seq-Gen: an application for the Monte Carlo simulation of DNA sequence evolution along phylogenetic trees.** *Computer Applications in the Biosciences* 1997, **13**(3):235-238.

15. Zhang J, Webb DM: **Evolutionary deterioration of the vomeronasal pheromone transduction pathway in catarrhine primates.** *Proc Nat Acad Sci* 2003, **100**(14):8337-8341.
